# Risk factors and prognostic significance of lateral pelvic lymph node dissection after neoadjuvant chemoradiotherapy for rectal patients with clinically suspected lateral lymph node metastasis

**DOI:** 10.1186/s12893-021-01443-5

**Published:** 2021-12-28

**Authors:** Sicheng Zhou, Yujuan Jiang, Wei Pei, Jianwei Liang, Zhixiang Zhou

**Affiliations:** Department of Colorectal Surgery, National Cancer Center/National Clinical Research Center for Cancer/Cancer Hospital, Chinese Academy of Medical Sciences and Peking Union Medical College, NO.17 Panjiayuannanli, Chaoyang District, Beijing, 100021 China

**Keywords:** Lateral pelvic lymph node dissection, Rectal cancer, Recurrence, Prognosis

## Abstract

**Aim:**

It is still controversial whether the addition of lateral pelvic lymph node (LPN) dissection (LPND) to total mesorectal excision (TME) can provide a survival benefit after neoadjuvant chemoradiotherapy (nCRT) in rectal cancer patients with pathological lateral lymph node metastasis (LPNM).

**Methods:**

Patients with clinically suspected LPNM who underwent nCRT followed by TME + LPND were systematically reviewed and divided into the positive LPN group (n = 15) and the negative LPN group (n = 58). Baseline characteristics, clinicopathological data and survival outcomes were collected and analysed.

**Results:**

Of the 73 patients undergoing TME + LPND after nCRT, the pathological LPNM rate was 20.5% (15/73). Multivariate analysis showed that a post-nCRT LPN short diameter ≥ 7 mm (OR 49.65; 95% CI 3.98–619.1; *P* = 0.002) and lymphatic invasion (OR 9.23; 95% CI 1.28–66.35; *P* = 0.027) were independent risk factors for pathological LPNM. The overall recurrence rate of patients with LPNM was significantly higher than that of patients without LPNM (60.0% vs 27.6%, *P* = 0.018). Multivariate regression analysis identified that LPNM was an independent risk factor not only for overall survival (OS) (HR 3.82; 95% CI 1.19–12.25; *P* = 0.024) but also for disease-free survival (DFS) (HR 2.33; 95% CI 1.02–5.14; *P* = 0.044). Moreover, N1-N2 stage was another independent risk factor for OS (HR 7.41; 95% CI 1.63–33.75; *P* = 0.010).

**Conclusions:**

Post-nCRT LPN short diameter ≥ 7 mm and lymphatic invasion were risk factors for pathological LPNM after nCRT. Furthermore, patients with pathological LPNM still show an elevated overall recurrence rate and poor prognosis after TME + LPND. Strict patient selection and intensive perioperative chemotherapy are crucial factors to ensure the efficacy of LPND.

## Statement

For patients with pathological LPNM, whether the employment of nCRT can reduce the local recurrence rate and improve survival is still not clear. The aim of this study was to identify risk factors for LPNM and investigate the oncological outcomes and prognostic values.

## Introduction

The lateral pelvic lymph node (LPN) is one of the common lymphatic metastasis areas of middle-low rectal cancer, and it has been reported that approximately 10–20% of rectal cancer patients with stage II-III disease develop LPN metastasis (LPNM) outside the field of total mesorectal excision (TME) [1, 2]. Most hospitals in Japan adopt a more active treatment attitude, and a prospective multicentre RCT (JCOG0212) conducted in Japan demonstrated that TME with ‘prophylactic’ LPN dissection (LPND) significantly decreased local recurrence rates compared with TME alone for patients who do not have LPN enlargement before surgery (7.4% vs 12.6%) [3]. In addition, the guidelines 2019 of the Japanese Society for Cancer of the Colon and Rectum recommend that TME + LPND should be performed if a preoperative or intraoperative diagnosis reveals the presence of LPNM [4]. However, recent literature has shown that, even with TME + LPND, patients with LPNM still show an increased risk of local recurrence and distant metastasis, resulting in a poor prognosis [5, 6]. In contrast, neoadjuvant chemoradiotherapy (nCRT) followed by TME was mostly employed for locally advanced rectal cancer, and several relevant randomized control studies revealed that, compared with TME alone, nCRT followed by TME could reduce the local recurrence rate by approximately 10% in clinical II or III rectal cancer patients [7, 8]. However, most of the patients in the above study had no LPNM, and the current opinion suggests that nCRT + TME without LPND is not sufficient for patients with enlarged LPN, with a lateral pelvic recurrence rate of 19.5% in patients with a LPN diameter greater than 7 mm [9–12].

In recent years, the literature has shown that TME plus selective LPND, according to indications after nCRT, can bring maximum therapeutic benefits to patients with suspicion of LPNM [13–16]. However, relevant studies on the prognostic factors of TME + LPND after nCRT in patients with suspicion of LPNM are few [17, 18]. In addition, for patients with pathological LPNM, whether the employment of nCRT can reduce the local recurrence rate and improve survival is still not clear. In the present study, patients with suspicion of LPNM based on preoperative images were selected, and all patients received TME + LPND after receiving nCRT. The aim of this study was to identify risk factors for LPNM and investigate the oncological outcomes and prognostic values for rectal cancer patients treated with TME + LPND following nCRT.

## Materials and methods

### Patients

A total of 83 low-middle rectal cancer patients with suspicion of LPNM who underwent TME + LPND after nCRT at the National Cancer Center/National Clinical Research Center for Cancer/Cancer Hospital, Chinese Academy of Medical Sciences and Peking Union Medical College between January 2015 and January 2021 were retrospectively collected and analysed. The inclusion criteria were as follows: (1) histologically confirmed adenocarcinoma; (2) lower tumour margin below the peritoneal reflection; and (3) suspicion of LPNM based on magnetic resonance imaging (MRI) evaluation. Patients with a previous history of other malignant tumours or achieved clinical complete response after nCRT and opted for watch and wait were excluded from this study. Finally, 73 patients met the above criteria and were included in the study. The ethics committee of the National Cancer Center/Cancer Hospital, Chinese Academy of Medical Sciences and Peking Union Medical College approved this study (NCC 2017-YZ-026, 17 October 2017). The study was conformed to the ethical standards of the World Medical Association Declaration of Helsinki and all methods were carried out in accordance with relevant guidelines and regulations. Prior written informed consent was obtained from all study participants.

### Diagnosis and treatment strategy

Lateral lymph node metastasis was evaluated by preoperative magnetic resonance imaging (MRI) in all patients, and the diagnostic criteria for LPNM were as follows: (1) short diameter of LPN > 8 mm; (2) inhomogeneous or intense enhancement; and (3) irregular shape with rough edges. Those patients meeting one or more of the above criteria can be diagnosed as having LPNM. Clinical stage and LPN status before and after treatment were evaluated according two imaging radiologists who specialized in colorectal cancer and the LPN short diameter was measured with electronic calipers on pelvic MRI. In addition, the two imaging radiologists who performed the preoperative evaluation were single-blind to patients information. The American Joint Committee on Cancer (AJCC, ninth edition) staging system was used for tumour staging. Lymph nodes in the area lying along the inferior mesenteric vessels, except LPN, were considered regional lymph nodes. All patients received nCRT, which consisted of a total radiation dose of 50 Gy (50 Gy/25 f/2 Gy) and oral capecitabine at a dose of 825 mg/m^2^ twice daily. TME + LPND was performed 6–8 weeks after completion of the last CRT. In present study, we decided to set 7 mm as the cut-off for post-nCRT LPN short diameter according to our previous research results and previous literature reports [14, 16].

### Surgical procedure

The LPND procedure (open or laparoscopic) was standardized, and unilateral or bilateral lymph node dissection was performed based on the location of the observed swollen LPN. The extent of LPLN includes internal iliac vessels (Region 263), external iliac vessels (Region 293), common iliac vessels (Region 273), and obturator lymph nodes (Region 283) that are distributed in the lateral pelvic area outside the pelvic plexus and hypogastric nerves [19].

### Follow-up

Follow-up was scheduled through telephone or outpatient visits every 3–6 months for the first 3 years and every 6 months thereafter until death due to recurrence or metastasis of rectal cancer or 1 February 2021, whichever came first. Tumour markers, computed tomography (CT), and pelvic MRI were examined during each follow-up. Local recurrence is defined as the recurrence of tumors with the same pathologic properties as the primary cancer at the site or surgical field after rectal cancer surgery, including anastomosis, mesentery, perineum, presacral tissue, genitourinary, pelvic lateral wall, etc.

### Statistical analysis

Analyses were performed using SPSS for Windows version 20.0 (SPSS, Chicago, Illinois, USA). Categorical variables and continuous variables are expressed as frequencies (percentages) and medians (ranges), respectively. Categorical variables were compared using the χ^2^ test or Fisher’s exact test. The statistically significant variables were included in multivariate logistic regression analysis, and odds ratios (ORs) and results were reported using hazard ratios (HRs) with 95% confidence intervals (95% CIs). Survival analysis, including overall survival (OS) and disease-free survival (DFS), was calculated using the Kaplan–Meier method, and univariate analysis was performed using the log-rank test. Factors with a *P* value < 0.05 in a univariate analysis were included in a multivariate analysis using the Cox proportional hazard model. A *P* value < 0.05 was considered to indicate statistical significance.

## Results

### Patient characteristics

Characteristics of all patients are shown in Table 1. A total of 73 patients were reviewed, with an average age of 55.8 years old, of which the majority were males (58.9%). The pathological type of most patients was moderately differentiated adenocarcinoma (80.8%). According to the AJCC staging system, 45 (61.6%) patients showed deep tumour infiltration (T3–T4), and 34 (46.6%) patients had regional lymph node metastasis (N1–N2). Twenty-three (31.5%) patients, 17 (23.3%) patients, and 16 (21.9%) patients developed perineural invasion, lymphatic invasion and vascular invasion, respectively. All 73 patients enrolled in the study underwent TME + LPND, 18 (24.7%) of whom underwent bilateral LPND. Fifteen (20.5%) patients had LPN pathologically confirmed, and the most common site of LPNM was the obturator region (12.3%), followed by the internal iliac vessels (6.8%), external iliac vessels (4.1%), and common iliac vessels (2.7%). The average operation time and intraoperative blood loss were 291.9 min and 87.3 ml, respectively. Postoperative complications occurred in 14 (19.2%) patients, and there was no perioperative death. The average number of harvested mesorectal lymph nodes and LPLNs was 15.4 and 9.0, respectively. The mean length of hospital stay after surgery was 8.7 days.

**Table 1:** Patient characteristics

Variables	Number (n = 73)
Age (years, mean ± SD) (range)	55.8 ± 10.4 (34–76)
Gender (%)	
Male	43 (58.9)
Female	30 (41.1)
BMI (kg/m^2^, mean ± SD)(range)	24.8 ± 3.2 (18.4–30.8)
Distance from AV (cm, mean ± SD)(range)	4.3 ± 2.0 (1–8)
Histology (%)	
Moderate	59 (80.8)
Poor/mucinous/signet	14 (19.2)
AJCC T stage (%)	
T1–T2	28 (38.4)
T3–T4	45 (61.6)
AJCC N stage (%)	
N0	40 (53.4)
N1–N2	34 (46.6)
Perineural invasion (%)	23 (31.5)
Lymphatic invasion (%)	17 (23.3)
Vascular invasion (%)	16 (21.9)
LPN metastasis (%)	15 (20.5)
Location of LPNM (%)	
Alongside the internal iliac vessel region	5 (6.8)
Alongside the external iliac vessel region	3 (4.1)
Alongside the obturator region	9 (12.3)
The common iliac vessel region	2 (2.7)
LPND (%)	
Unilateral dissection	55 (75.3)
Bilateral dissection	18 (24.7)
Mesorectal lymph nodes harvested(range)	15.4 ± 8.0 (8–58)
LPLNs harvested(range)	9.0 ± 5.5 (5–38)
Operative time (min, mean ± SD)(range)	291.9 ± 69.4 (170–480)
Estimated intraoperative blood loss (ml, mean ± SD)(range)	87.3 ± 103.7 (10–300)
Postoperative complications (%)	14 (19.2)
Postoperative hospital days (days, mean ± SD)(range)	8.7 ± 4.8 (6–44)
Adjuvant chemotherapy	54 (74.0)

### Univariate and multivariate logistic regression analysis of LPNM

The univariate analysis is shown in Table 2. A post-nCRT LPN short diameter of 7 mm was the cut-off point in our institution. A total of 93.3% of patients with LPNM had a post-nCRT LPN short diameter ≥ 7 mm, which was higher than that of patients without LPNM. Patients with LPNM showed deep tumour infiltration (86.7% vs 55.2%, *P* = 0.025) and were more likely to develop lymphatic invasion (53.3% vs 15.5%, *P* = 0.006) than those without LPNM. In addition, patients with LPNM had a higher proportion of adverse pathological types (40.0% vs 13.8%, *P* = 0.027). The above significant variables in univariate analysis were included in multivariate analysis, and the results showed that post-nCRT LPN short diameter ≥ 7 mm (OR 49.65; 95% CI 3.98–619.1; *P* = 0.002) and lymphatic invasion (OR 9.23; 95% CI 1.28–66.35; *P* = 0.027) were identified as independent risk factors for pathological LPNM (Table 3).

**Table 2:** Univariate analysis of LPNM for 73 patients after nCRT

Variables	Positive LPN (n = 15)	Negative LPN (n = 58)	P
Gender			0.493
Male	10 (66.7)	33 (56.9)	
Female	5 (33.3)	25 (43.1)	
Age (years)			0.786
≤ 60	9 (60.0)	37 (63.8)	
> 60	6 (40.0)	21 (36.2)	
Distance from the AV (cm)			0.923
< 5	9 (60.0)	34 (58.6)	
5–10	6 (40.0)	24 (41.4)	
Post-nCRT LPN short diameter (mm)			< 0.001
< 7	1 (6.7)	44 (75.9)	
≥ 7	14 (93.3)	14 (24.1)	
LPN intensity			0.141
Normal	7 (46.7)	39 (67.2)	
Inhomogeneous or intense enhancement	8 (53.3)	19 (32.8)	
LPN shape			0.046
Normal	5 (33.3)	36 (62.1)	
Irregular shape or rough edges	10 (66.7)	22 (37.9)	
Pre-nCRT CEA level (ng/ml)			
< 5	12 (80.0)	49 (84.5)	0.979
≥ 5	3 (20.0)	9 (15.5)	
ypT stage			0.025
T1–2	2 (13.3)	26 (44.8)	
T3–4	13 (86.7)	32 (55.2)	
ypN stage			0.080
N0	5 (33.3)	34 (58.6)	
N1–2	10 (66.7)	24 (41.4)	
Mesorectal LN metastasis	3.4 ± 6.2	1.4 ± 2.5	0.151
Histology			0.027
Moderate	9 (60.0)	50 (86.2)	
Poor/Mucinous/signet adenocarcinoma	6 (40.0)	8 (13.8)	
Lymphatic invasion	8 (53.3)	9 (15.5)	0.006
Perineural invasion	7 (46.7)	16 (27.6)	0.269
Vascular invasion	5 (33.3)	11 (19.0)	0.396
Types of operation (%)			0.771
Low anterior resection	6 (40.0)	20 (34.5)	
Abdominoperineal resection	8 (53.3)	36 (62.1)	
Hartmann procedure	1 (6.7)	2 (3.4)	
LPND type (%)			1.000
Unilateral dissection	11 (73.3)	44 (75.9)	
Bilateral dissection	4 (26.7)	14 (24.1)	
Adjuvant chemotherapy	13 (86.6)	41 (70.7)	0.296

*SD* standard deviation, *BMI* body mass index, AV anal verge, *LPN*, lateral pelvic lymph node, *LPNM* lateral pelvic lymph node metastasis, *LPND* lateral pelvic lymph node dissection

**Table 3:** Multivariate analysis of LPNM for 73 patients after nCRT

Variables	OR	95%CI	*P*
Poor/Mucinous/signet adenocarcinoma	2.83	0.41–19.66	0.294
Post-nCRT LPN short diameter ≥ 7 mm	49.65	3.98–619.1	0.002
ypT3–4	2.93	0.36–23.73	0.314
Lymphatic invasion	9.23	1.28–66.35	0.027
Irregular LPN shape or rough edges	2.73	0.49–15.36	0.254

*AV* anal verge, *LPN* lateral pelvic lymph node, *LN* lymph node, *LPNM* lateral pelvic lymph node metastasis, *LPND* lateral pelvic lymph node dissection

### Postoperative Recurrence and Survival Analysis

The median follow-up period of the whole group was 28.0 (range 2–66) months. In total, 15 (20.5%) patients died, and 25 (34.2%) patients developed local recurrence or distant metastasis during follow-up. The postoperative overall recurrence rate of patients with LPNM was higher than that of patients without LPNM (60.0% vs 27.6%, *P* = 0.018). Although the local recurrence rate (26.7% vs 6.9%, *P* = 0.085) and distant metastasis rate (40.0% vs 22.4%, *P* = 0.292) were higher in patients with LPNM, the difference was not statistically significant (Table 4).

**Table 4:** ^Postoperative recurrence of 73 patients with or without pathological LPNM^

	Positive LPN (n = 15)	Negative LPN (n = 58)	*P*
Overall recurrence (%)	9 (60.0)	16 (27.6)	0.018
Local recurrence	4 (26.7)	4 (6.9)	0.085
Distant metastasis	6 (40.0)	13 (22.4)	0.292
Liver metastasis	4 (26.7)	7 (12.1)	
Lung metastasis	1 (6.7)	6 (10.3)	
Bone metastasis	1 (6.7)	2 (3.4)	
Peritoneal metastasis	1 (6.7)	0 (0)	
Others	0 (0)	1 (1.7)	

*LPN* lateral pelvic lymph node, *LPNM* lateral pelvic lymph node metastasis, node dissection

**Fig. 1:**
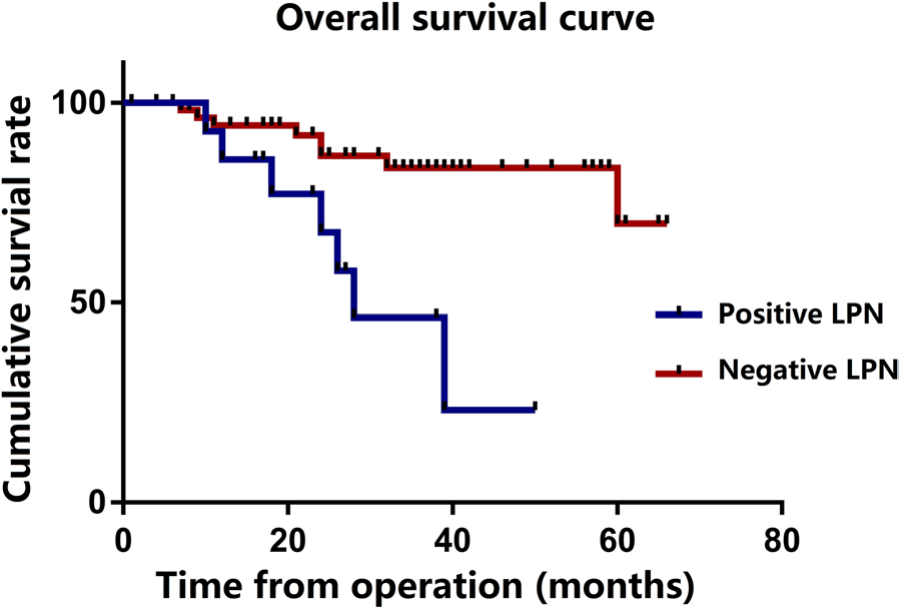
Overall survival of 73 patients with or without pathological LPNM

**Fig. 2:**
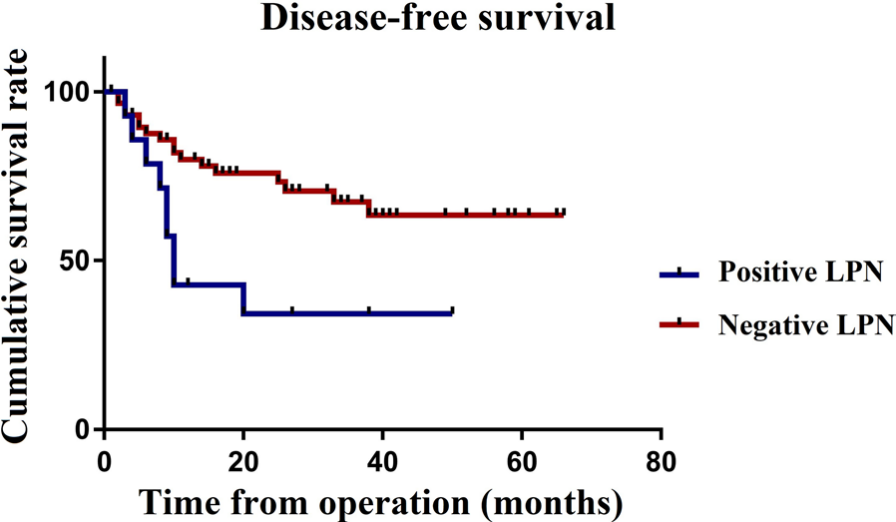
Disease-free su survival of 73 patients with or without pathological LPNM

The Kaplan curves showed that the OS (*P* = 0.006) and DFS (*P* = 0.018) of patients with LPNM were significantly worse than those of patients without LPNM (Figs. 1 and 2). The 1-, 2- and 3-year OS rates were 85.7% vs. 94.3%, 67.5% vs. 86.7% and 46.3% vs. 83.7% in patients with or without LPNM, respectively. The 1-, 2- and 3-year DFS rates were 42.9% vs. 79.9%, 34.3% vs. 75.9% and 34.3% vs. 67.3% in patients with or without LPNM, respectively (Table 5).

**Table 5:** Overall survival and disease-free survival of 73 patients with or without pathological LPNM

	N	Overall survival			Disease-free survival		
		1-year	2-year	3-year	1-year	2-year	3-year
LPN status							
Positive LPN	15	85.7%	67.5%	46.3%	42.9%	34.3%	34.3%
Negative LPN	58	94.3%	86.7%	83.7%	79.9%	75.9%	67.3%

*LPN* lateral pelvic lymph node, *LPNM* lateral pelvic lymph node metastasis, node dissection

Univariate and multivariate regression analyses were conducted to identify prognostic factors for OS and DFS of patients who underwent TME + LPND. Univariate analysis showed that adverse pathological types, N1–N2 stage, and LPNM were associated with poor OS (*P* < 0.05). In addition, DFS was significantly affected by the pre-nCRT CEA level, N1–N2 stage and LPNM (*P* < 0.05). Multivariate regression analysis identified that LPNM was an independent risk factor not only for OS (HR 3.82; 95% CI 1.19–12.25; *P* = 0.024) but also for DFS (HR 2.33; 95% CI 1.02–5.14; *P* = 0.044. Moreover, N1–N2 stage was another independent risk factor for OS (HR 7.41; 95% CI 1.63–33.75; *P* = 0.010) (Table 6).

**Table 6:** Univariate and multivariate analyses for overall survival and disease-free survival of the 73 rectal patients with clinical LPNM who underwent TME + LPND

Variables	Overall survival				Disease-free survival			
	Univariate analysis		Multivariate analysis		Univariate analysis		Multivariate analysis	
	HR(95%CI)	P	HR(95%CI)	P	HR(95%CI)	P	HR(95%CI)	P
Gender: male/female	1.01 (0.36–2.83)	0.993			1.19 (0.54–263)	0.660		
Age	1.03 (0.98–1.08)	0.314			1.00 (0.97–1.04)	0.953		
BMI (Kg/m^2^)	1.02 (0.86–1.21)	0.801			0.95 (0.83–1.08)	0.404		
Pre-nCRT CEA level	1.01 (0.99–1.03)	0.495			1.01 (1.00–1.03)	0.026	1.01 (0.97–1.03)	0.262
Pre-nCRT CA19-9 level	1.01 (0.99–1.01)	0.093			1.00 (0.99–1.01)	0.158		
Post-nCRT CEA level	1.09 (0.95–1.26)	0.220			1.04 (0.95–1.13)	0.399		
Post-nCRT CA19-9 level	1.01 (0.99–1.01)	0.135			1.01 (0.99–1.01)	0.183		
Histology	3.50 (1.24–9.89)	0.018	2.21 (0.75–6.48)	0.150	1.81 (0.95–3.29)	0.076		
Distance from AV	1.16 (0.91–1.50)	0.238			1.01 (0.81–1.23)	0.939		
T stage: T3–4/T1–2	3.41 (0.70–8.00)	0.109			2.22 (0.89–5.52)	0.087		
N stage: N1–2/N0	8.00 (1.80–35.54)	0.006	7.41 (1.63–33.75)	0.010	2.41 (1.05–4.93)	0.044	2.04 (0.93–5.10)	0.087
LPN metastasis	4.42 (1.54–12.74)	0.006	3.82 (1.19–12.25)	0.024	2.67 (1.18–6.02)	0.018	2.33 (1.02–5.14)	0.044
Mesorectal LN harvested	1.02 (0.98–1.07)	0.299			1.02 (0.98–1.01)	0.388		
LPN harvested	1.01 (0.94–1.09)	0.742			1.01 (0.96–1.06)	0.730		
Operative time	1.00 (0.99–1.01)	0.980			1.00 (0.99–1.01)	0.635		
Estimated bleeding	1.00 (0.99–1.01)	0.682			1.00 (1.00–1.01)	0.089		
Postoperative complications	0.73 (0.20–2.67)	0.633			0.84 (0.34–2.10)	0.714		

*BMI* body mass index, *AV* anal verge, *LPN,* lateral pelvic lymph node, *LN* lymph nodes, *LPNM* lateral pelvic lymph node metastasis, *LPND* lateral pelvic lymph node dissection, *TME* total mesorectal excision

## Discussion

Previous studies from Eastern and Western countries have reported that, for patients with preoperative lateral lymph node enlargement, nCRT without LPND results in a high rate of lateral pelvic recurrence after surgery [20–22]. Therefore, we conducted a retrospective study to explore the surgical indications for LPND after nCRT for patients with suspected LPNM before surgery and to investigate the oncological outcomes and prognostic values for rectal cancer patients treated with TME + LPND following nCRT. Our results preliminarily found indications of LPND after nCRT in patients with low-middle rectal cancer, and that the current treatment strategy for LPNM is inadequate. Therefore, the purpose of this study was to explore the causes of poor prognosis in patients with LPNM and to explore ways to potentially improve treatment strategies.

In previous studies, the incidence of LPNM for patients with stage II-III rectal cancer was demonstrated to be 10–20% [1, 2]. All the patients included in the present study were suspected of having LPNM by preoperative MRI evaluation, and the rate of pathological LPNM was only 20.5%, which is related to the relatively loose imaging diagnostic criteria of LPNM. The present study showed that pathological LPNM was significantly affected by post-nCRT size and lymphatic invasion. The selection of the optimal cut-off value of post-nCRT LPN size remains controversial, with the most common cut-off values currently being 5 mm [1, 23, 24] and 7 mm [14, 16]. Chen et al. reported that persistent LPN size ≥ 7 mm on post-nCRT MRI was significantly associated with LPNM after nCRT (OR 7.539, 95% CI 1.49–38.21; *P* = 0.015) [16]. Furthermore, Inoue et al., however, thought that 7 mm could be a more appropriate cut-off [14]. In our study, we set 7 mm as the cut-off for post-nCRT LPN size. As a result, 93.3% (14/15) and 24.1% (14/58) of patients with LPN short diameter ≥ 7 mm were found in the positive and negative LPN groups, respectively. Multivariate logistic regression analysis demonstrated that a post-nCRT LPN short diameter ≥ 7 mm (OR 49.65; 95% CI 3.98–619.1; *P* = 0.002) and lymphatic invasion (OR 9.23; 95% CI 1.28–66.35; *P* = 0.027) were independent risk factors for pathological LPNM, and these results were consistent with the above mentioned results. Rectal lymphatic circumfluence can be divided into three directions: upward, lateral and downward. The lateral rectal ligament is rich in blood vessels and lymph nodes, which is considered as a lymphatic pathway between low rectum and lateral region. Therefore, the occurrence of LPNM should be highly vigilant in patients with lymphatic invasion, and adjuvant therapy should be strengthened during perioperative period while complete resection of the lateral rectal ligament.

A meta-analysis of 18 studies involving 6133 patients suggested that additional LPND results in greater postoperative morbidity, urinary dysfunction, and sexual dysfunction, without improving recurrence and long-term survival [25]. Moreover, it has been reported that patients with pathological LPNM, even after TME + LPND, still have a higher local recurrence rate and a worse prognosis than patients without pathological LPNM [5, 6, 26–28]. However, the role of nCRT in LPNM has become clearer in recent years, and this study investigated the oncology outcomes of TME + LPND after nCRT for patients with LPNM. Our study demonstrated that patients with pathological LPNM who received TME + LPND after nCRT still showed a higher overall recurrence rate after surgery (60.0% vs 27.6, *P* = 0.018). Similarly, a retrospective study involving 899 patients at a high-volume cancer centre in Japan conducted by Wang et al. revealed that, even with LPND, patients with pathological LPNM still showed an elevated risk of local recurrence (30.0% vs 10.0, *P* = 0.025) [5]. Meanwhile, the present study revealed that the 3-year OS (46.3% vs 83.7%, *P* = 0.006) and DFS (34.3% vs 67.3%, *P* = 0.018) of patients with LPNM were significantly worse than those without LPNM, and the multivariate regression analysis identified that LPNM was an independent risk factor not only for OS (HR 3.82; 95% CI 1.19–12.25; P = 0.024) but also for DFS (HR 2.33; 95% CI 1.02–5.14; P = 0.044) through multivariate regression analysis. A retrospective study involving 149 rectal patients conducted by Sato et al. showed that the 5-year OS rate was significantly worse in patients with LPNM (36.2% vs 69.8%, *P* = 0.0004), and multivariate Cox regression analysis for factors affecting the prognosis showed that LLN metastases had an independent predictive value in determining prognosis (HR 2.41; 95% CI 1.37–4.26; *P* = 0.002) [28], which is basically consistent with our results. Considering the greater postoperative morbidity, urinary dysfunction, and sexual dysfunction associated with LPND, we suggest that it is necessary to explore which types of patients with pathological LPNM can benefit from TME + LPND after nCRT.

Our study found that even if LPND was performed after nCRT, the prognosis of patients with LPNM was often poor. Moreover, it should be noted that the results of present study showed that 1-year DFS in patients with LPNM was only 42.9%, lower than previous literature reports [29]. It may be because at the time of initial diagnosis, micro-metastases of the liver and lungs could not be detected or identifiable. A retrospective study conducted by Hiyoshi et al. also revealed that the prognosis of rectal cancer patients with LPNM is poor, not only in the overall rectal cancer patient population but also in patients with stage IV disease [26]. Therefore, for patients with clinical LPNM, the adequate preoperative examination should be fully evaluated for distant metastasis, and postoperative adjuvant systemic chemotherapy should be strengthened to eliminate micro-metastasis. In addition, several literature demonstrated that LPND appears to confer survival benefits to certain patients with single LPN involvement in the obturator region or internal iliac vessel region [30, 31]. Therefore, appropriate patient selection are also important factors to ensure the efficacy of LPND.

This study was associated with several limitations, including the retrospective nature and small sample size of only 73 patients included. Second, the study period was from 2015 to 2021, and the adjuvant chemotherapy regimens adopted by the included patients were inconsistent, which may cause some interference with the prognosis. Secondly, in this study, the DFS of patients with LPNM was only 35.7% in 1 year after surgery, which may be related to inadequate preoperative diagnosis and inadequate treatment strategy. Therefore, the present study does not deny the efficacy of LPND after nCRT, only emphasizes that for patients with LPNM, adequate preoperative evaluation should be carried out, strictly in accordance with LPND indications, to avoid execution in stage IV patients. Moreover, this study only verified the oncology outcomes of LPND for patients with clinically suspected LPNM. It is impossible to compare the local control effect of prophylactic LPND in stage II-III patients with middle-low rectal cancer due to the nonroutine practice of prophylactic LPND in our institution. Finally, the number of patients with pathological LPNM in our analysis was too small to perform an appropriate multivariate analysis to identify which types of patients with LPNM may achieve a survival benefit from LPND. Therefore, randomized controlled studies with larger numbers of patients are needed to further verify our findings.

## Conclusion

In conclusion, post-nCRT LPN short diameter ≥ 7 mm and lymphatic invasion were risk factors for pathological LPNM after nCRT. Furthermore, patients with pathological LPNM still show an elevated overall recurrence rate and poor prognosis after TME + LPND. Strict patient selection and intensive perioperative chemotherapy are crucial factors to ensure the efficacy of LPND.

## Data Availability

The data that support the fndings of this study are available on reasonable request from the corresponding author. The data are not publicly available due to privacy and ethical restrictions.

## Abbreviations

TME: Total mesorectal excision
LPND: Lateral pelvic lymph node dissection
nCRT: Neoadjuvant chemoradiotherapy
LPNM: Lateral lymph node metastasis
DFS: Disease-free survival
OS: Overall survival
MRI: Magnetic resonance imaging
AJCC: American Joint Committee on Cancer

## References

[1] Wang P, Zhou S, Zhou H (2019). Evaluating predictive factors for determining the presence of lateral pelvic node metastasis in rectal cancer patients following neoadjuvant chemoradiotherapy. Colorectal Dis.

[2] Kanemitsu Y, Komori K, Shida D (2017). Potential impact of lateral lymph node dissection (LLND) for low rectal cancer on prognoses and local control: a comparison of 2 high-volume centers in Japan that employ different policies concerning LLND. Surgery.

[3] Fujita S, Mizusawa J, Kanemitsu Y (2017). Mesorectal excision with or without lateral lymph node dissection for clinical stage II/III lower rectal cancer (JCOG0212): a multicenter, randomized controlled, noninferiority trial. Ann Surg.

[4] Hashiguchi Y, Muro K, Saito Y, Japanese Society for Cancer of the Colon and Rectum (2020). Japanese Society for Cancer of the Colon and Rectum (JSCCR) guidelines 2019 for the treatment of colorectal cancer. Int J Clin Oncol.

[5] Wang L, Hirano Y, Heng G (2020). The significance of lateral lymph node metastasis in low rectal cancer: a propensity score matching study. J Gastrointest Surg.

[6] Numata M, Tamagawa H, Kazama K (2021). Lateral lymph node dissection for mid-to-low rectal cancer: is it safe and effective in a practice-based cohort?. BMC Surg.

[7] Bosset JF, Collette L, Calais G (2006). Chemotherapy with preoperative radiotherapy in rectal cancer. N Engl J Med.

[8] Sauer R, Becker H, Hohenberger W (2004). Preoperative versus postoperative chemoradiotherapy for rectal cancer. N Engl J Med.

[9] Schaap DP, Ogura A, Nederend J (2018). Prognostic implications of MRI-detected lateral nodal disease and extramural vascular invasion in rectal cancer. Br J Surg.

[10] Perez RO, São Julião GP, Vailati BB (2018). Lateral node dissection in rectal cancer in the era of minimally invasive surgery: a step-by-step description for the surgeon unacquainted with this complex procedure with the use of the laparoscopic approach. Dis Colon Rectum.

[11] Malakorn S, Ouchi A, Sammour T (2018). Robotic lateral pelvic lymph node dissection after neoadjuvant chemoradiation: view from the West. Dis Colon Rectum.

[12] Ogura A, Konishi T, Cunningham C (2019). Neoadjuvant (chemo)radiotherapy with total mesorectal excision only is not sufficient to prevent lateral local recurrence in enlarged nodes: results of the multicenter lateral node study of patients with low cT3/4 rectal cancer. J Clin Oncol.

[13] Akiyoshi T, Matsueda K, Hiratsuka M (2015). Indications for lateral pelvic lymph node dissection based on magnetic resonance imaging before and after preoperative chemoradiotherapy in patients with advanced low rectal cancer. Ann Surg Oncol.

[14] Inoue Y, Saigusa S, Hiro J (2016). Clinical significance of enlarged lateral pelvic lymph nodes before and after preoperative chemoradiotherapy for rectal cancer. Mol Clin Oncol.

[15] McCawley N, Clancy C, O'Neill BD (2016). Mucinous rectal adenocarcinoma is associated with a poor response to neoadjuvant chemoradiotherapy: a systematic review and meta-analysis. Dis Colon Rectum.

[16] Chen JN, Liu Z, Wang ZJ (2020). Selective lateral lymph node dissection after neoadjuvant chemoradiotherapy in rectal cancer. World J Gastroenterol.

[17] Tomono A, Yamashita K, Kanemitsu K (2016). Prognostic significance of pathological response to preoperative chemoradiotherapy in patients with locally advanced rectal cancer. Int J Clin Oncol.

[18] Ogura A, Akiyoshi T, Nagasaki T (2017). Feasibility of laparoscopic total mesorectal excision with extended lateral pelvic lymph node dissection for advanced lower rectal cancer after preoperative chemoradiotherapy. World J Surg.

[19] Hashiguchi Y, Muro K, Saito Y (2020). Japanese Society for Cancer of the C and Rectum: Japanese society for cancer of the colon and rectum (JSCCR) guidelines 2019 for the treatment of colorectal cancer. Int J Clin Oncol.

[20] Kim TG, Park W, Choi DH (2014). Factors associated with lateral pelvic recurrence after curative resection following neoadjuvant chemoradiotherapy in rectal cancer patients. Int J Colorectal Dis.

[21] Kim MJ, Kim TH, Kim DY (2015). Can chemoradiation allow for omission of lateral pelvic node dissection for locally advanced rectal cancer?. J Surg Oncol.

[22] Kusters M, Slater A, Muirhead R (2017). What to do with lateral nodal disease in low locally advanced rectal cancer? A call for further reflection and research. Dis Colon Rectum.

[23] Oh HK, Kang SB, Lee SM (2014). Neoadjuvant chemoradiotherapy affects the indications for lateral pelvic node dissection in mid/low rectal cancer with clinically suspected lateral node involvement: a multicenter retrospective cohort study. Ann Surg Oncol.

[24] Yamaoka Y, Kinugasa Y, Shiomi A (2017). Preoperative chemoradiotherapy changes the size criterion for predicting lateral lymph node metastasis in lower rectal cancer. Int J Colorectal Dis.

[25] Hajibandeh S, Hajibandeh S, Matthews J (2020). Meta-analysis of survival and functional outcomes after total mesorectal excision with or without lateral pelvic lymph node dissection in rectal cancer surgery. Surgery.

[26] Hiyoshi Y, Miyamoto Y, Kiyozumi Y (2020). Risk factors and prognostic significance of lateral pelvic lymph node metastasis in advanced rectal cancer. Int J Clin Oncol.

[27] Kim JC, Takahashi K, Yu CS (2007). Comparative outcome between chemoradiotherapy and lateral pelvic lymph node dissection following total mesorectal excision in rectal cancer. Ann Surg.

[28] Sato H, Maeda K, Maruta M (2011). Prognostic significance of lateral lymph node dissection in node positive low rectal carcinoma. Int J Colorectal Dis.

[29] Nagasaki T, Akiyoshi T, Fujimoto Y (2017). Preoperative chemoradiotherapy might improve the prognosis of patients with locally advanced low rectal cancer and lateral pelvic lymph node metastases. World J Surg.

[30] Yokoyama S, Takifuji K, Hotta T (2014). Survival benefit of lateral lymph node dissection according to the region of involvement and the number of lateral lymph nodes involved. Surg Today.

[31] Akiyoshi T, Watanabe T, Miyata S (2012). Results of a Japanese nationwide multi-institutional study on lateral pelvic lymph node metastasis in low rectal cancer: is it regional or distant disease?. Ann Surg.

